# Glue, Onyx, Squid or PHIL? Liquid Embolic Agents for the Embolization of Cerebral Arteriovenous Malformations and Dural Arteriovenous Fistulas

**DOI:** 10.1007/s00062-021-01066-6

**Published:** 2021-07-29

**Authors:** Dominik F. Vollherbst, René Chapot, Martin Bendszus, Markus A. Möhlenbruch

**Affiliations:** 1 Department of Neuroradiology, Heidelberg University Hospital, INF 400, 69120 Heidelberg, Germany; 2 Neuroradiology, Alfried-Krupp-Krankenhaus Rüttenscheid, Essen, Germany

**Keywords:** Interventional neuroradiology, Vascular malformation, Hemorrhagic stroke, Embolization, Embolic agents

## Abstract

**Background:**

Endovascular embolization is an effective treatment option for cerebral arteriovenous malformations (AVMs) and dural arteriovenous fistulas (DAVFs). A variety of liquid embolic agents have been and are currently used for embolization of AVMs and DAVFs. Knowledge of the special properties of the agent which is used is crucial for an effective and safe embolization procedure.

**Material and Methods:**

This article describes the properties and indications of the liquid embolic agents which are currently available: cyanoacrylates (also called glues), and the copolymers Onyx, Squid and PHIL, as well as their respective subtypes.

**Results:**

Cyanoacrylates were the predominantly used agents in the 1980s and 1990s. They are currently still used in specific situations, for example for the occlusion of macro-shunts, for the pressure cooker technique or in cases in which microcatheters are used that are not compatible with dimethyl-sulfoxide. The first broadly used copolymer-based embolic agent Onyx benefits from a large amount of available experience and data, which demonstrated its safety and efficacy in the treatment of cerebral vascular malformations, while its drawbacks include temporary loss of visibility during longer injections and artifacts in cross-sectional imaging. The more recently introduced agents Squid and PHIL aim to overcome these shortcomings and to improve the success rate of endovascular embolization. Novelties of these newer agents with potential advantages include extra-low viscosity versions, more stable visibility, and a lower degree of imaging artifacts.

**Conclusion:**

All the available liquid embolic agents feature specific potential advantages and disadvantages over each other. The choice of the most appropriate embolic agent must be made based on the specific material characteristics of the agent, related to the specific anatomical characteristics of the target pathology.

## Introduction

Endovascular embolization is an effective treatment option for vascular malformations of the brain, such as cerebral arteriovenous malformations (AVMs) and dural arteriovenous fistulas (DAVFs) [1, 2]. For AVMs, endovascular embolization can be performed either alone or in combination with microsurgical resection and radiosurgery. In the case of a multimodal approach, endovascular embolization is most commonly performed as the first treatment modality [3]. After one or more embolization sessions, subsequent microsurgical resection or radiosurgery can be performed. For most DAVFs, endovascular embolization is currently regarded as the first-line treatment due to high occlusion rates and low complication rates, compared to other treatment modalities [4].

The principle of endovascular embolization is occlusion of the pathological blood vessels of the underlying vascular malformation using a microcatheter, which is selectively positioned within or proximal to the pathology, by injection of an occlusive substance into these vessels. Currently, nearly all materials which are used for endovascular embolization of cerebral AVMs and DAVFs are liquid embolic agents (LEAs) [1, 2, 4, 5]. Particulate embolic agents, such as polyvinyl alcohol (PVA) particles, were occasionally used in earlier years, but were virtually completely replaced by LEAs in the last decades due to high recurrence rates [6]. Coils are sometimes used as an adjunct to LEA embolization for specific techniques, such as the pressure cooker technique or for the selective occlusion of mostly venous structures of DAVFs [7–10]. LEAs that are available for the embolization of cerebral vascular malformations can basically be divided into two groups: cyanoacrylates or adhesive embolic agents, which have a glue-like behavior (colloquially they are often named glue) and copolymers or nonadhesive embolic agents, which are described to feature lava-like or rubber-like characteristics.

The differentiation between so-called adhesive and non-adhesive embolic agents refers to a potential complication of cyanoacrylates, which is the inability to retrieve a microcatheter when its tip is trapped in highly concentrated acrylic glue. This presumably underreported complication was the initiator for the development of the non-adhesive embolic agent ethylene vinyl alcohol (EVOH or EVAL), which aimed to avoid this issue [11]. EVOH diluted in dimethyl sulfoxide (DMSO) appeared to be significantly less adhesive and led to the development of Onyx (Medtronic, Irvine, CA, USA); however, to allow the injection of higher volumes of embolic agent into a vascular malformation, the way to inject LEAs was further developed under the influence of Jacques Moret who developed the “plug and push” technique which requires trapping the tip of the microcatheter [12, 13]. Similar to the embolization with cyanoacrylates, this led to an inability to retrieve the microcatheter as soon as the length of reflux exceeded a certain distance or the duration of trapping exceeded several minutes. The high rate of stuck microcatheters or ruptured vessels after forceful withdrawal when using a so-called non-adhesive agent led to the development of microcatheters with a detachable tip, as initiated by the Sonic microcatheter (Balt, Montmorency, France) [14]. A classification based on the chemical structure of LEAs seems therefore preferable than a classification based on the adhesiveness.

For both of these two groups, different substances with several subclasses, each with individual properties are currently available on the market. Of these substances, some were introduced only recently aiming to improve the efficacy and safety of endovascular embolization. The aim of this article is to systematically describe the most frequently used LEAs which are currently available for the endovascular treatment of cerebral vascular malformations and to summarize available experimental and clinical data, the differences between these agents and their specific characteristics.

## Cyanoacrylates

### Material Characteristics

**Fig. 1:**
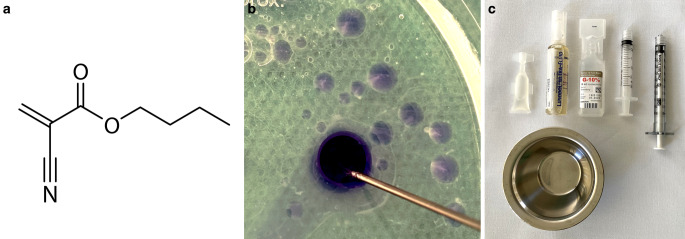
Chemical formula (**a**), macroscopic appearance (**b**) and material for the injection of cyanoacrylates (**c**). The structural chemical formula of n-butyl cyanoacrylate (nBCA) is shown exemplarily. After the injection into saline, n-BCA (Histoacryl shown here for visualization purposes because of its blue color) spreads out in droplets. The copolymers immediately begin to polymerize, which is visualized by the whitish smaller droplets and by the whitish margins of the larger droplets. Cyanoacrylates are commonly delivered in small plastic bottles (Glubran 2 is shown here). After manual mixing with iodized oil in a small bowl, and after flushing the microcatheter with glucose solution, the cyanoacrylate-iodized oil mixture is ready for injection

The chemical formula, macroscopic appearance, and material for the injection of cyanoacrylates is illustrated in Fig. 1. In their liquid form, cyanoacrylates consist of monomer molecules. In the presence of blood (specifically hydroxide ions) these molecules rapidly undergo an exothermic chain-growth polymerization with temperatures rising up to 80–90 °C, resulting in an adhesive material, which occludes the embolized blood vessel. Chemically, cyanoacrylates consist of cyanoacrylate ester molecules, which basically differ in the length of the alkyl chains.

### Available Cyanoacrylates

Differing in the molecular structure and specific additives, there are several acrylic agents available for endovascular embolization, which are summarized in Table 1.

**Table 1:** Overview of cyanoacrylates

Product name	Manufacturer	Component(s)	Special characteristics
Histoacryl	B. Braun, Melsungen, Germany	nBCA^a^	No official approval for intravascular use, but historically the most used agent
Glubran 2	GEM SRL, Viareggio, Italy	nBCA + MS^b^	Extended polymerization time and reduced inflammatory effect by the additive MS, only available in Europe
Magic glue^c^	Balt, Montmorency, France	nHCA	Reduced adhesive strength to the microcatheter, only available in Europe
TruFill	Cerenovus, Irvine, CA, USA	nBCA	Only available in the USA
Fuaile	Fuaile, Beijing, China	nBCA and 2‑octyl cyanoacrylate (OCA)	Only available in China

^a^*nBCA* n-butyl cyanoacrylate, ^b^*MS* metacryloxysulpholane

^c^Magic glue was formerly commercially available as Purefill (Péters Surgical, Bobigny Cedex, France)

The first field of application of cyanoacrylates in medicine was wound closure. Initially, only isobutyl-2-cyanoacrylate (IBCA; Ethicon, Somerville, MA, USA), also called bucrylate, was used and available for endovascular embolization. In the late 1980s, IBCA was no longer available and was consequently replaced by n-butyl cyanoacrylate (nBCA), manufactured and distributed as Histoacryl by B. Braun (Melsungen, Germany). Histoacryl has been widely used for the embolization of a variety of vascular disorders, including cerebral vascular malformations, such as AVMs and DAVFs, for more than 40 years [5, 15–17]. Histoacryl is the only acrylic agent with an additional coloring agent allowing clear identification of the syringe containing the embolic agent. Despite its widespread use until today, Histoacryl is still not approved for intravascular use. The lack of an official approval and the desire to improve the embolic properties led to the development of new cyanoacrylates.

Glubran 2 (GEM SRL, Viareggio, Italy) is composed of nBCA and metacryloxysulpholane (MS). The co-monomer MS lowers the polymerization temperature to about 45 °C aiming to reduce the inflammatory and cytotoxic effects, and prolongs the polymerization time of the embolic agent aiming to prolong the duration of injection. The potential benefits of this co-monomer mixture were investigated in two animal studies, but to date have not been systematically assessed in clinical studies [18, 19]. A clinical study, compromising 31 patients with cerebral AVMs, demonstrated that Glubran 2 can be used for the curative embolization of AVMs with a complete angiographic occlusion rate of 87.1%, an overall complication rate of 19.4% and a permanent disabling neurological deficit rate of 0% [20].

Magic glue (Balt, Montmorency, France), formerly known as Purefill (Péters Surgical, Bobigny Cedex, France), is another cyanoacrylate with potential benefits over other products. Magic glue is n-hexyl cyanoacrylate (nHCA), a monomer which has an occlusive efficacy similar to Histoacryl and Glubran 2, while possessing a very low adhesive strength, according to two experimental preclinical studies [21, 22]. The lower adhesive strength allows for prolonged injections up to 1h without restrictions in the ability to remove the microcatheter. A potential drawback is the risk of delayed dislodgement of the embolic agent, which has been seldom reported.

Trufill, which is currently distributed by Cerenovus (Irvine, CA, USA), and only available in the USA, is very similar to Histoacryl, consisting also of nBCA. Trufill is distributed together with a vial containing 1 g of tantalum powder, which can be added to the embolic mixture to ensure better visibility. According to the manufacturer, the addition of 0.5 g of tantalum powder is advised at higher cyanoacrylate concentrations (> 50%). In a recently published study, reporting on 251 patients who were treated for sequential flow reduction of AVMs prior to definitive surgery or radiosurgery, embolization using Trufill was performed with a low overall morbidity (major embolization-related morbidity: 7.2%) and mortality (overall mortality: 1.6%) [23].

Fuaile (Fuaile, Beijing, China), composed of 2‑octyl cyanoacrylate (OCA) and nBCA, is another co-monomer which is used for endovascular embolization, but is currently only commercially available in China. To date, no studies investigating Fuaile for the embolization of cerebral vascular malformations have been published.

### Polymerization Speed and Visibility

Cyanoacrylates cannot be used in pure form for embolization as the microcatheter would occlude immediately due to instant polymerization of the embolic agent within the microcatheter. Therefore, cyanoacrylates are combined with iodized oil (Lipiodol, Guerbet, Villepinte, France or formerly Ethiodol, Savage Laboratories, Melville, NY, USA). The main goal of iodized oil is to reduce the speed of polymerization. The higher the volume of the admixed iodized oil, the slower the polymerization speed of the mixture. The ratio between cyanoacrylate and iodized oil usually varies between 1:1 (50% cyanoacrylate, 50% iodized oil) and 1:4 (20% cyanoacrylate, 80% iodized oil) for the embolization of cerebral vascular malformations, depending on several factors, such as the position of the microcatheter, the diameter of the embolized vessel, the velocity of blood flow, the intended duration of injection and consequently also the amount of embolic agent to be injected. For the occlusion of macro-shunts, for example, the tip of the microcatheter is positioned at the level of the shunt and a 1:1 mixture is injected. To occlude a larger part of an AVM or a DAVF, or if a microcatheter cannot be navigated to a distal position, the catheter can be placed in a more proximal position, and a ratio of 1:4 is used, whereby the potential risk of the occlusion of normal arteries must be considered. The second goal of iodized oil is to enable a sufficient visibility of the embolic agent, as cyanoacrylates are not radiopaque and cannot therefore be seen under fluoroscopy. For macro-shunts, where only a minimal amount of iodized oil is used, tantalum powder may be added to allow better visualization [24].

To prevent polymerization within the microcatheter with subsequent obstruction of it, the catheter has to be flushed with 5–10% glucose solution before the injection of cyanoacrylates.

Reflux of LEA back along the catheter tip should be avoided or be tolerated only for a short distance and a very short period of time, since the larger area of contact to the microcatheter can substantially increase the risk of catheter entrapment. Despite adequate preflushing and the addition of iodized oil, the catheter through which the embolization is performed has to be removed after a short period of time, except with Magic Glue. Quick removal and manual aspiration on the syringe used for the embolization can reduce the risk of non-target embolization.

Most microcatheters developed before the introduction of the copolymer-based LEAs are not compatible with DMSO, which is an obligatory component of these agents. A frequently used microcatheter in AVM treatment is the Magic® (Balt). It is still the most navigable microcatheter in distal locations. Of the available LEAs, only cyanoacrylates can be injected through the Magic, even though in our personal experience, a short time injection with copolymer-based LEAs may be performed through the Magic in specific indications; however, this is expressly not recommended by the manufacturers.

### Clinical Data

For many years, cyanoacrylates were the only available LEAs for the embolization of cerebral vascular malformations.

For the treatment of cerebral AVMs during this time, cyanoacrylates were most frequently used for preoperative embolization, embolization prior to radiosurgery or for the selective embolization of rupture sites. Consequently, in most of these studies in the era before the introduction of copolymer-based embolic agents, complete occlusion of the AVM was often not the primary aim of the endovascular treatment [25–28]. Several further studies demonstrated the efficacy and safety of cyanoacrylates for AVM embolization [23, 29]. After the introduction of Onyx, several studies, including a prospective, randomized trial and a meta-analysis, compared the embolization of AVMs using these two agents [5, 30, 31]. The abovementioned meta-analysis, published by Elsenousi et al. in 2014, found that embolization using cyanoacrylates tends to be associated with lower permanent complication rates (poor neurological outcome: 5.2% for nBCA vs. 6.8% for Onyx), while the angiographic cure rate was higher for Onyx (complete occlusion rate: 13.7% for nBCA vs. 30.5% for Onyx) [5]. Currently, while most interventionalists use Onyx or other copolymer-based embolic agents for the treatment of cerebral AVMs, cyanoacrylates are still used effectively for special applications, such as the embolization of high-flow shunts or for specific techniques, such as the pressure cooker technique [7, 8]. For the embolization of spinal vascular malformations, cyanoacrylates are still commonly and effectively used [32].

For DAVFs, similarly to AVM embolization, cyanoacrylates were effectively and safely used as embolic agents for many years [33–35]. The endovascular occlusion rates varied between 30% and 71% in these studies. Since the introduction of Onyx, cyanoacrylates were replaced for most applications and the complete occlusion rates of DAVFs could substantially be improved, concentrating around 80% in the current literature [2, 4]; however, similar to AVMs, cyanoacrylates are still effective in the case of high-flow shunt embolization or for the pressure cooker technique, which can also be applied for the treatment of DAVFs.

## Copolymers

### Available Copolymers

The currently available and most frequently used copolymers, which are used for the embolization of cerebral vascular malformations are Onyx (Medtronic, Irvine, CA, USA), Squid (Balt, Montmorency, France) and PHIL (MicroVention, Aliso Viejo, CA, USA). Of these three agents, only Onyx has received both CE and FDA approval and is therefore available in Europe and in the USA, while Squid and PHIL are currently not yet available in the USA. Characteristics of these embolic agents are summarized in Table 2.

**Table 2:** Overview of copolymers

Product name	Manufacturer	Component(s)	Radiopaque component	Formulations^a^	Special characteristics
Onyx	Medtronic, Irvine, CA, USA	EVOH copolymer, micronized tantalum powder, DMSO	Tantalum	18, 20, 34	Long experience, efficacy and safety demonstrated in multiple studies
Squid	Balt, Montmorency, France	EVOH copolymer, micronized tantalum powder, DMSO	Tantalum	12, 12LD, 18, 18LD, 34, 34LD	Smaller grain size of tantalum powder compared to Onyx
Menox	Meril Life Sciences, Gujarat, India	EVOH copolymer, micronized tantalum powder, DMSO	Tantalum	18, 20, 34	Similar to Onyx
PHIL	MicroVention, Aliso Viejo, USA	Polylactide-co-glycolide, polyhydroxyethyl-methacrylate, triiodophenol, DMSO	Triiodophenol (covalently bound to the copolymers)	LV, 25%, 30%, 35%	No preparation needed, lower degree of imaging artifacts

*EVOH* ethylene vinyl alcohol, *DMSO* dimethyl-sulfoxide, *PHIL* precipitating hydrophobic injectable liquid, *LD* low density, *LV* low viscosity, ^a^numbers indicate the viscosity in centipoise for Onyx and Squid and the concentration of the copolymer for PHIL

### Embolic Properties

Despite their structural differences, Onyx, Squid and PHIL feature similar embolic properties, which are addressed in this section. Unique properties of the respective agents are explained in the respective subsections.

Compared with cyanoacrylates, which are relatively hard in their solid form, the copolymer-based embolic agents feature a more plastic state. All of these three copolymers occlude blood vessels by precipitation, a mechanism which has been compared with the hardening of lava flow from a volcano. The solidification results from the dissipation of DMSO, a solvent which is a component of all these embolic agents, into the blood. Before the injection of the embolic agent, the microcatheter has to be flushed with DMSO, which prevents precipitation of the LEA within the microcatheter (similar to glucose for cyanoacrylates). DMSO can cause local toxic effects on the embolized blood vessels, potentially resulting in vasospasms, inflammation of the vessel wall or angionecrosis [36, 37]. Furthermore, DMSO can seldom cause bradycardia or even asystole, caused by the trigeminocardiac reflex [38, 39]. A very slow injection during flushing and also during the embolization can reduce these effects. Since DMSO can damage the synthetic material of the devices which are used for the injection, only DMSO-resistant catheters and syringes should be used.

While cyanoacrylates polymerize within a period of several seconds to a few minutes, the mechanism of precipitation can take many minutes, depending on the size of the embolized blood vessels and the velocity of blood flow within them. This aspect allows for more controlled embolization, longer injection times and consequently better forward penetration and filling of the target malformation but also facilitates unwanted diffusion into normal arteries. Microcatheters with detachable tips are to be used whenever prolonged injections are planned and retraction on the microcatheter could potentially cause a damage as in cerebral AVMs. The use of detachable tip microcatheters is generally not required in the treatment of DAVFs. The most frequently used detachable tip microcatheters are Sonic (Balt) and Apollo (Medtronic). Using detachable tip microcatheters, reflux of LEA up to the detachment site can be tolerated, and the microcatheter can be removed from the body, while the detached tip stays within the embolized blood vessel [14, 40–43]. For all kinds of microcatheters (detachable tip or nondetachable tip) retrieval of the microcatheter is usually performed by slowly pulling it for a distance of a few centimeters, while aspirating on the catheter using a syringe. The traction force and the narrowing of the microcatheter’s outer diameter caused by the traction is sufficient for successful and safe microcatheter retrieval in the majority of cases.

While reflux of embolic agent back along the catheter can substantially increase the risk of catheter entrapment during the injection of cyanoacrylates, reflux is part of the embolization technique when dealing with copolymers. The injection of copolymer-based LEAs is most commonly performed using 1 mL syringes applying the so-called plug and push technique [12, 13]. The formation of a plug around the catheter tip, consisting of hardened LEA, resulting after several injections of LEA with a certain amount of intended or tolerated reflux, is often a prerequisite for effective penetration or complete embolization of an AVM or DAVF [44, 45]. Reflux is however poorly controllable. Whenever it occurs, one can just stop the injection, expecting that the embolic agent will diffuse towards the malformation at the next injection. If not, excessive reflux can lead to the occlusion of non-target arteries, possibly leading to ischemic stroke [44–46]. Frequently, the injection has to be paused several times until effective penetration of the vascular malformation is achieved; however, this is not always successful. In some cases, for example due to an unfavorable position of the microcatheter tip, a sufficient diffusion into the vascular malformation may not be obtained. In such cases, it can be necessary to leave the compartment untreated to prevent inducing ischemia due to excessive reflux.

To reduce the risk or amount of reflux and increase the chance of diffusion into the vascular malformation, an antireflux mechanism, such as a plug created by coils together with cyanoacrylates (the pressure cooker technique) or a dual-lumen balloon catheter can be used [7, 47, 48].

### Onyx

#### Material Characteristics

**Fig. 2:**
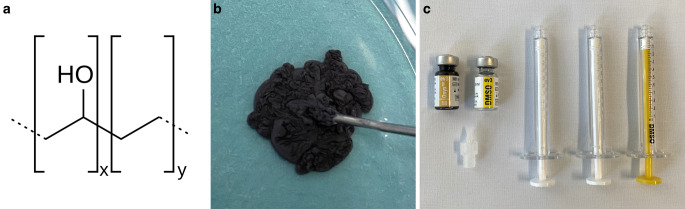
Chemical formula (**a**), macroscopic appearance (**b**) and material for the injection of Onyx (**c**). The structural chemical formula of ethylene vinyl alcohol (EVOH) copolymer is shown. After injection into saline, Onyx flows outwardly like lava, while solidifying from outside to inside (Onyx 18 shown here). Onyx is delivered in 1.5 mL vials together with a vial of dimethyl-sulfoxide (DMSO) and with DMSO-compatible syringes. After shaking of 20 min or longer and flushing the microcatheter with DMSO, Onyx can be injected via the syringe-catheter interface adapter

The chemical formula, macroscopic appearance and material for the injection of Onyx are illustrated in Fig. 2. Onyx is composed of three components: EVOH copolymer, micronized tantalum powder and DMSO. EVOH copolymer is the active substance which solidifies after the dissipation of DMSO. The micronized tantalum powder enables the radiopacity of the embolic agent. The package includes a 1.5 mL vial of Onyx and DMSO, respectively, as well as three DMSO-compatible syringes. The Onyx vial has to be kept on a shaker for at least 20 min prior to its use to ensure homogeneous suspension of the tantalum particles. Either no shaking or insufficient shaking may lead to inadequate visualization of Onyx during the injection. A syringe-catheter interface adapter should be used to avoid precipitation of Onyx within the hub of the microcatheter and to reduce the dead space of the system.

#### Formulations

Onyx is available in three different formulations: Onyx 18, Onyx 20 and Onyx 34. The numbers indicate the viscosity in centipoise (cps) at 40 °C. Accordingly, Onyx 18 features the lowest viscosity, while Onyx 20 is highly viscous and Onyx 34 is extra-highly viscous. For comparison, pure nBCA has a viscosity of approximately 10 cps (the addition of iodized oil influences the viscosity). The viscosity of the EVOH/DMSO mixture is not linearly related to the concentration of EVOH: Onyx 18 is composed of 6% EVOH/94% DMSO, Onyx 20 of 6.5% EVOH/93.5% DMSO and Onyx 34 of 8% EVOH/92% DMSO. Onyx 18 is the most commonly used formulation for the embolization of AVMs and DAVFs while the high viscosity formulations are typically used for larger arterial feeders and high-flow shunts.

#### Visibility

The visibility of Onyx is caused by the admixed tantalum powder. After sufficient (> 20 min) shaking, Onyx features excellent x‑ray visibility; however, after some minutes the tantalum particles begin to sediment within the syringe and within the microcatheter, which can impair the visibility of Onyx during longer embolization procedures or lead to occlusion of the microcatheter.

A well-known drawback of the EVOH-based LEAs is the production of imaging artifacts in conventional and cone-beam computed tomography (CT) imaging [49–52]. These artifacts can represent a crucial obstacle for the detection of peri-procedural or postprocedural hemorrhage, and impede an adequate treatment planning of a subsequent radiosurgery [53–58].

#### Embolic Properties

Observations during AVM and DAVF embolization procedures showed that the precipitation of Onyx occurs from the outside to the inside, resulting in a harder circular outer portion and a softer or liquid inner phase. This issue has to be considered during image-guided embolization, since a blood vessel which is completely opacified during an embolization procedure might not be embolized completely. The wall of the opacified vessel might already by lined with LEA, while there is still a perfused central lumen. Not knowing this phenomenon might cause incomplete embolization of certain structures. Moreover, an opacified healthy brain-supplying blood vessel which is aimed to be preserved, might still be perfused and supply its respective brain parenchyma.

#### Clinical Data

Of all available copolymer-based LEAs, Onyx was the first introduced and most described. The very first use of an EVOH-based LEA, the precursor substance of Onyx, was published by Taki in 1994 [11]. In its current form, Onyx is available in Europe since 1999 after receiving the CE mark, while in the USA, it was approved for endovascular embolization in 2005.

Regarding cerebral AVM embolization, multiple studies have demonstrated its efficacy and safety for the endovascular embolization of cerebral vascular malformations since its introduction [1, 5, 30, 31, 59–62]. As indicated in the literature section for cyanoacrylates, several studies showed that Onyx can be advantageous over cyanoacrylates for the treatment of cerebral AVMs [5, 30, 31]. One of the largest studies which reported the endovascular treatment of AVMs with Onyx is that of Saatci et al. in 2011, compromising 350 patients with 27% presenting with high-grade AVMs [61]. Using a prolonged injection technique, a complete occlusion of the AVM could be obtained in 51% and high-grade AVMs that were otherwise not treatable were amenable to radiosurgery. Another cornerstone publication on AVM embolization is the BRAVO study, a prospective, multicenter study which evaluated the safety and efficacy of Onyx in cerebral AVM embolization [1]. In 117 patients, complete occlusion using embolization alone was obtained in 24% of patients, while complementary treatment, mostly radiosurgery, was performed in 82%, being accompanied by an acceptable procedure-related morbidity rate of 5% and mortality rate of 4%.

For the treatment of cranial DAVF, one of the largest studies is that published by Gross et al. in 2016 [2]. They analyzed a cohort of 251 patients and compared the treatment outcomes between patients treated before (87 patients) and during the Onyx era (173 patients, not all of them were treated with Onyx). They observed a significantly higher rate of initial angiographic occlusion during the Onyx era (60% vs. 76%). A further large study, published in 2019, reported the results of DAVF embolization with Onyx as the primary embolic agent in 104 patients, demonstrating a high occlusion rate of 91% and a low permanent complication rate of 2% [63]. A meta-analysis of transarterial Onyx embolization, which included 19 studies involving 426 patients, showed similar results with a high pooled initial complete occlusion rate of 82% and low pooled procedure-related complication rates (neurologic deficits: 4%, morbidity: 3%, mortality: 0%) [4].

### Squid

#### Material Characteristics

**Fig. 3:**
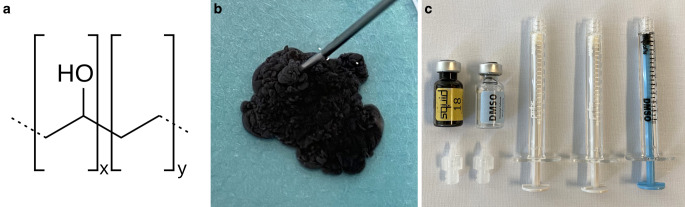
Chemical formula (**a**), macroscopic appearance (**b**) and material for the injection of Squid (**c**). The structural chemical formula of ethylene vinyl alcohol (EVOH) copolymer is shown. After injection into saline, similarly to Onyx, Squid features a lava-like behavior, also solidifying from outside to inside (Squid 18 shown here). Squid is delivered in 1.5 mL vials together with a vial of dimethyl-sulfoxide (DMSO), with DMSO-compatible syringes and two syringe-catheter interface adapters. After shaking of 20 min or longer and flushing the microcatheter with DMSO, Squid can be injected

The chemical formula, macroscopic appearance and material for the injection of Squid are illustrated in Fig. 3. Squid has the same components as Onyx: EVOH copolymer, micronized tantalum powder and DMSO. The essential difference with Squid is the smaller size of the grains of tantalum powder. This smaller grain size aims to enhance the homogeneity in radiopacity and improve the visibility during longer injections times [64]. A package of Squid includes one 1.5 mL vial of Squid and DMSO, respectively, as well as three DMSO-compatible syringes and two syringe-catheter interface adapters. Squid also has to be kept on a shaker for at least 20 min prior to its use.

#### Formulations

Squid is available in six different formulations: Squid 12, Squid 12LD, Squid 18, Squid 18LD, Squid 34 and Squid 34LD (numbers indicate the viscosity in cps at 40 °C). The main innovations of Squid are the extra-low viscosity versions Squid 12/Squid 12LD and the low density (LD) variants. The extra-low viscosity version, which has a lower concentration of EVOH, was developed to achieve deeper penetration of the LEA into a vascular malformation. The LD variants contain 30% less tantalum and are consequently less radiopaque while featuring the same embolic properties as the standard versions. This modification is thought to enable a better assessment of the embolized AVM vasculature and the volume of injected LEA. After partial Onyx embolization, especially of larger AVMs and DAVFs, the embolic agent cast is often very radiopaque, which can impede the visibility of residual AVM compartments. This drawback could be improved with the LD variants of Squid. The usage of the LD variants could help to differentiate the LEA which is being injected from “older” embolic agents and improve the visibility of LEA through a partially embolized nidus or fistula network which superimposes the region which is embolized. To date, no study has investigated the advantages regarding visibility of the Squid LD versions.

#### Visibility

An experimental study showed that Squid has a significantly slower drop in radiopacity compared to Onyx, caused by the slower sedimentation of the extra-small tantalum particles [64]. This allows for a better visibility of Squid, especially during longer procedures.

Similar to Onyx, Squid can cause severe artifacts in conventional CT and cone-beam CT with potential negative influences on the detection of periprocedural and postprocedural hemorrhage and/or subsequent radiosurgery. An experimental in vitro study by Pop et al. demonstrated a lower degree of artifacts for all variants of Squid, compared with Onyx 18 [50]. These findings, however, could not be confirmed in a more recent and more comprehensive experimental work by Schmitt et al. who only observed minor differences between Squid and Onyx regarding their artifacts in CT imaging [51]. Both studies showed that the LD variants cause less artifacts compared to the standard versions, which can be attributed to the lesser amount of tantalum within the LEA.

#### Embolic Properties

Compared with Onyx, there are far fewer studies for Squid reporting on its embolic properties. Based on the similar components, precipitation of Squid is also thought to occur from the outside to the inside with the same abovementioned impacts on the embolization procedure.

The extra-low viscosity versions Squid 12 and Squid 12LD feature special embolic properties which are quite different from Onyx 18. Due to its lower viscosity, Squid 12 can enable faster and longer antegrade flow of LEA during embolization, and accordingly lead to a faster and more effective penetration of the target vasculature. Two experimental studies, focusing on the extra-low viscosity version of PHIL (explained in more detail below), used Squid 12 as a comparator and showed that the embolization using the extra-low viscous LEAs Squid 12 and PHIL LV can be more effective than the standard viscous versions in terms of a higher embolization extent and fewer reflux events [65, 66]. These findings were also described in a few clinical studies; however, further studies are needed to further analyze potential advantages and disadvantages of Squid 12 and Squid 12LD [65–67].

According to the manufacturer, the embolic properties do not differ between the LD and the standard variants of Squid. To date this issue has not yet been scientifically investigated.

#### Clinical Data

Clinical data on the embolization of AVMs and DAVFs with Squid are rare. Akmangit et al. reported their preliminary single-center experience with Squid in 2014, including the treatment of 16 AVMs and 9 DAVFs [68]. According to their preliminary results, Squid is a safe and effective embolic agent for treatment of cerebral AVMs and DAVFs. A study reporting on the usage of Squid 12 for the embolization of DAVFs, compromising 20 embolization procedures in 19 patients, achieved a high complete angiographic occlusion rate (17/19 patients), without any major periprocedural adverse events [67]. They stated that the lower viscosity seems to allow an easier penetration of the LEA into the DAVF vasculature. Further clinical studies on Squid are needed.

### PHIL

#### Material Characteristics

**Fig. 4:**
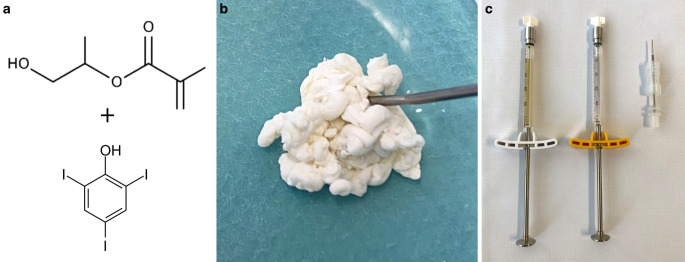
Chemical formula, macroscopic appearance and material for the injection of PHIL. The structural chemical formula of polyhydroxyethylmethacrylate is shown exemplarily, as well as triiodophenol, which is covalently bound to the copolymers. After injection into saline, PHIL flows eccentrically and solidifies rather column-like (PHIL 25% shown here). PHIL is delivered in a ready to use 1.0 mL syringe, together with a syringe of DMSO and a syringe-catheter interface adapter

The chemical formula, macroscopic appearance and material for the injection of PHIL are illustrated in Fig. 4. PHIL (precipitating hydrophobic injectable liquid) has a composition different to Onyx and Squid. It consists of two copolymers (polylactide-co-glycolide and polyhydroxyethylmethacrylate) as its active components and triiodophenol (an iodine compound), the latter being covalently bound to the two copolymers, enabling radiopacity of the agent. PHIL also uses DMSO as solvent. A PHIL package consists of two prefilled syringes containing 1 mL of PHIL and DMSO, respectively, and a syringe-catheter interface adapter. PHIL is ready to use and does not need to be shaken or prepared otherwise before its use.

#### Formulations

PHIL is available in four different formulations: PHIL LV, PHIL 25%, PHIL 30% and PHIL 35%, of which PHIL 25% is the standard low viscosity version and PHIL LV is the extra-low viscosity version. The numbers indicate the concentration of the two abovementioned copolymers. Similar to the extra-low viscous Squid 12, PHIL LV aims to improve the penetration of the LEA into an AVM nidus or a DAVF vascular network. The difference between PHIL LV and PHIL 25% is the length of the polymer chains which are shorter for PHIL LV, causing the lower viscosity. The concentration of the copolymers, and accordingly also the concentration of iodine, and thus the radiopacity, are technically the same for PHIL 25% and PHIL LV.

#### Visibility

As explained above, sedimentation can disturb the visibility of Onyx and Squid, especially during longer interventions. Since iodine, as the radiopaque component, is covalently bound to the copolymers, PHIL features a constant radiopacity over time.

Three experimental works, one in vivo study and two in vitro studies, investigated the imaging artifacts of PHIL [49, 51, 52]. They demonstrated that PHIL induces only minor artifacts in conventional CT and cone-beam CT imaging, significantly less CT artifacts compared to Onyx and Squid, and no artifacts in magnetic resonance imaging (MRI).

#### Embolic Properties

Experimental in vitro and in vivo works studied the embolic properties of PHIL 25% and demonstrated that they are similar to those of Onyx [69, 70]. Two further experimental in vivo studies focused on PHIL LV [65, 66]. Both of them observed a tendency towards higher embolization extents for PHIL LV compared to Squid 12 and Onyx 18; however, these results could not yet be confirmed in clinical studies.

**Fig. 5:**
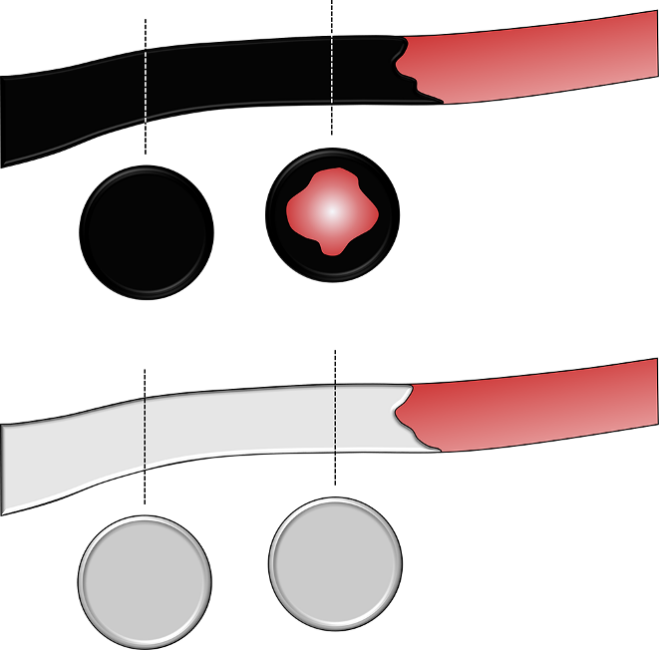
Schematic illustration of the embolic behavior of EVOH-based LEAs and PHIL. Compared with the EVOH-based LEAs Onyx and Squid (illustrated as black material), which feature a rather lava-like behavior, with solidification from the outside to the inside, PHIL (illustrated as white material) solidifies and moves rather like a column. This phenomenon has to be considered when assessing opacified blood vessels during embolization. For Onyx and Squid, an opacified blood vessel might still be centrally perfused, and consequently not completely embolized, while for PHIL, an opacified blood vessel should be completely occluded. These observations, however, are based on early clinical reports and experimental studies and were not yet systematically investigated

Compared with Onyx and Squid, PHIL is thought to flow forward rather like a column, and less like the aforementioned lava-like behavior with precipitation from the outside to the inside, which is illustrated in Fig. 5; however, to date this phenomenon has not been systematically analyzed.

PHIL features a relatively high embolic capacity. Comparing PHIL 25% and Onyx 18, lower volumes of PHIL are required for the same extent of embolization [70]. This finding was observed in an in vitro study but to date has not been assessed in further studies.

#### Clinical Data

There are more scientific data on PHIL than on Squid, however, compared with Onyx, the quantity of scientific data is still limited.

To date, AVM embolization using PHIL was the focus of four clinical studies, of which all included only a limited number of patients [71–74]. These studies, which altogether included not more than 50 patients, demonstrated that PHIL is effective and safe for cerebral AVM embolization.

For the treatment of DAVFs using PHIL a few studies reported their initial experience with it [74–77]. These studies reported that PHIL can be an effective and safe alternative to EVOH-based LEAs. The largest study was published by Lamin et al. in 2017 who analyzed 26 patients treated with PHIL [76]. They achieved an occlusion rate of 77% which is comparable to Onyx, while only 1 adverse event was observed.

## Discussion

In the last decades, there have been substantial advances in the development of embolic agents for the treatment of cerebral vascular malformations. The most frequently used LEAs are cyanoacrylates (as aforementioned, also called glues), Onyx, Squid and PHIL, as well as their respective subtypes. Cerebral AVMs and cranial DAVFs are the two entities, for which especially LEAs play an important role as the most frequently used embolic agents for the endovascular treatment. Every one of these agents features unique properties and potential advantages over other agents. To achieve a safe and effective endovascular embolization of a cerebral vascular malformation, the treating interventionalist should be familiar with the specific characteristics of these various agents which are used for the procedure.

The use of cyanoacrylates, which were the predominantly applied LEAs for AVM and DAVF embolization for a long period of time, has decreased in the last decades, while they are currently still used effectively in specific situations [7, 8, 28]. Besides their embolic properties, a potential advantage of cyanoacrylates over the copolymers is their price: in most countries, cyanoacrylates are much cheaper than Onyx, Squid or PHIL. Different subtypes of cyanoacrylates from different manufacturers are available, while the choice in most countries is limited due to restricted regional availability. Specific subtypes may be advantageous in specific situations, for example the lower vasotoxicity of Glubran 2 or the reduced adhesive strength of Magic glue to the microcatheter; however, to date these specific features have not been sufficiently explored scientifically.

Based on their composition and the available data, the currently available copolymers-based LEAs have potential advantages over each other. A clear advantage of Onyx is the large range of scientific data and the long experience with this material, which is by far greater than what is currently known about the newer copolymers Squid and PHIL. Single studies with > 300 patients, and reviews with > 5000 patients for AVMs, and with > 100 patients (single studies) and > 400 patients (reviews) for DAVFs are available which analyzed and demonstrated the safety and efficacy of Onyx for the embolization of these entities [2, 4, 61, 63, 78].

The smaller grain size of the tantalum powder of Squid reduces the speed of sedimentation and enables more homogeneous visibility, compared to Onyx, which is especially relevant during longer embolization procedures; however, during very long interventions, the tantalum nevertheless sediments. PHIL uses covalently bound iodine for its visibility, which does not sediment and accordingly enables a constant homogeneous visibility. These material characteristics can be taken into consideration if longer embolization is expected.

An advantage of both Squid and PHIL is the availability of an extra-low viscosity version: Squid 12 and PHIL LV. LEAs with extra-low viscosity could improve the success rate for these entities. Experimental and clinical studies showed that these extra-low viscous versions are feasible, effective and safe [65–68]. Experimental studies in the porcine rete mirabile, serving as an AVM model also showed that these LEAs can achieve better results than Onyx 18 in terms of a higher embolization extent and fewer reflux events [65, 66]. Despite these potential advantages, the lower viscosity can also bear some risks: less effective proximal plug formation, inadvertent embolization of nontarget arteries (e.g. supplying healthy brain tissue or cranial nerves) and premature distal embolization, potentially leading to premature occlusion of draining veins [65, 67]. Because of the potentially less effective proximal plug formation, proximal flow control by using a dual-lumen balloon catheter or the pressure cooker technique seems to be reasonable when using these extra-low viscosity LEAs. Clinical studies which systematically compared these new extra-low viscosity versions with standard versions are currently not yet available.

A well-known drawback of copolymer-based LEAs is the induction of imaging artifacts which occur predominantly in CT imaging [49, 50]. Since especially AVMs but also DAVFs are associated with an increased risk of periprocedural and postprocedural hemorrhage, these artifacts can impede the detection of a perilesional hematoma during or after embolization [53]. Furthermore, a considerable number of AVMs cannot be completely treated by endovascular means, which is why additional radiosurgery is often required afterwards [54]. The planning recordings which are essential for stereotactic radiosurgery are based on conventional CT imaging, which is why embolization-related artifacts can represent another significant drawback for adequate planning of a radiosurgical treatment after previous embolization [55, 56]. PHIL produces significantly less artifacts compared to Onyx and Squid, which might improve the detection of periprocedural or postprocedural hemorrhages and be advantageous for a subsequent radiosurgical treatment [49, 51]. The few available clinical studies on PHIL reported the low degree of artifacts, but to date no study has investigated the influence of the different types of LEA on the detection of periprocedural/postprocedural hemorrhage or a following radiotherapy.

A further potential advantage of PHIL is that it does not need any kind of preparation before its use. Cyanoacrylates have to be mixed with iodized oil, and Onyx and Squid have to be kept on a shaker for at least 20 min before their use. In an emergency situation, PHIL as a ready to use product can be immediately used for the embolization of, for example, a site of hemorrhage.

Vasotoxic and inflammatory effects on the embolized vessels can be caused by all of the abovementioned LEAs [79–82]. For the cyanoacrylates, the degree of toxicity is comparably higher and can be attributed to the cyanoacrylates themselves, while for the copolymer-based LEAs, the toxic effects are often mild and are mainly caused by the admixed DMSO. For cyanoacrylates, the reactions are caused by the exothermic polymerization reaction, and by the release of highly toxic components such as acryl acetate and formaldehyde [82]. The perioperative administration of anti-inflammatory drugs, such as corticosteroids, can prevent or reduce the development of embolization-related inflammatory reactions [16].

The EVOH-based LEAs appear black due to the admixed tantalum powder, which can lead to macroscopically visible black colorization of superficially embolized blood vessels, for example branches of the occipital artery after DAVF embolization. PHIL appears white in its solid state, and accordingly does not cause this tattoo effect, which can be of advantage if superficial vessels are embolized. Also owing to the tantalum content, Onyx and Squid can occasionally cause hazards during a subsequent surgery, since bipolar cautery near an embolized vessel can lead to sparking and combustion [83]. This phenomenon was not described for cyanoacrylates or for PHIL.

In summary, all of the available LEAs can feature specific advantages and disadvantages over each other. Most of these potential advantages, especially those of the more recently introduced materials, need further investigation in experimental and clinical trials. Adequate knowledge of the special properties of the agent which is used is crucial for an effective and safe embolization procedure. None of the available LEAs is suitable for all indications. The choice of the most appropriate LEA has to be made based on the specific material characteristics of the particular LEA, related to the specific anatomical characteristics of the target pathology (for example: size of the blood vessels, velocity of blood flow and level of obstruction).
